# Evaluation of Psychological Resources of Young Adults With Type 1 Diabetes Mellitus During the Transition From Pediatric to Adult Diabetes Clinics: Multicenter Cross-sectional Study

**DOI:** 10.2196/46513

**Published:** 2023-05-29

**Authors:** Katarzyna Cyranka, Anna Juza, Hanna Kwiendacz, Katarzyna Nabrdalik, Janusz Gumprecht, Maciej Małecki, Tomasz Klupa, Bartłomiej Matejko

**Affiliations:** 1 Department of Metabolic Diseases Jagiellonian University Medical College Kraków Poland; 2 Clinic of Metabolic Diseases University Hospital in Kraków Kraków Poland; 3 Department of Psychiatry Jagiellonian University Medical College Kraków Poland; 4 Diabetes Outpatient Clinic Clinical Provincial Hospital of Frederic Chopin No. 1 in Rzeszów Rzeszów Poland; 5 College of Medical Sciences University of Rzeszow Rzeszów Poland; 6 Department of Internal Medicine, Diabetology and Nephrology Faculty of Medical Sciences in Zabrze Medical University of Silesia Katowice Poland

**Keywords:** young adults, type 1 diabetes, transitioning care, psychological, diabetes, cross-sectional study, anxiety, socioeconomic, validation, anger, depression, outpatient, chronic disease, pediatric, adulthood, coping mechanism

## Abstract

**Background:**

The transition period of patients with type 1 diabetes from pediatric to adult-oriented health care is associated with poorer glycemic control and less frequent clinic attendance. Fears and anxiety about the unknown, care approach differences in adult settings, and sadness about leaving the pediatric provider all contribute to a patient’s reluctance to transition.

**Objective:**

This study aimed to evaluate the psychological parameters of young patients with type 1 diabetes transitioning to an adult outpatient clinic during the first visit.

**Methods:**

We examined 50 consecutive patients (n=28, 56% female) transitioning from March 2, 2021, to November 21, 2022, into adult care (3 diabetes centers from 3 regions in southern Poland: A, n=16; B, n=21; and C, n=13) and their basic demographic information. They completed the following psychological questionnaires: State-Trait Anxiety Inventory (STAI), Generalized Self-Efficacy Scale, Perceived Stress Scale, Satisfaction with Life Scale, Acceptance of Illness Scale, Multidimensional Health Locus of Control Scale Form C, Courtauld Emotional Control Scale, and Quality of Life Questionnaire Diabetes. We compared their data with those for the general healthy population and patients with diabetes from Polish Test Laboratory validation studies.

**Results:**

During the first adult outpatient visit, patients’ mean age was 19.2 (SD 1.4) years, with a diabetes duration of 9.8 (SD 4.3) years and BMI of 23.5 (SD 3.1) kg/m^2^. Patients came from diverse socioeconomic backgrounds: 36% (n=18) live in villages, 26% (n=13) live in towns with ≤100,000 inhabitants, and 38% (n=19) live in bigger cities. Regarding therapy type, 68% (n=34) were treated with insulin pump therapy, whereas 32% (n=16) were treated with multiple daily injections. Patients from center A had a mean glycated hemoglobin level of 7.5% (SD 1.2%). There was no difference regarding the level of life satisfaction, perceived level of stress, and state anxiety between the patients and reference populations. Patients had similar health locus of control and negative emotions control to the general population of patients with diabetes. Most patients (n=31, 62%) believe that control over their health depends on themselves, whereas 52% (n=26) believe that it depends mostly on others. Patients had higher levels of suppression of negative emotions—anger, depression, and anxiety—than the age-matched general population. Additionally, the patients were characterized by a higher acceptance of illness and higher level of self-efficacy compared to the reference populations: 64% (n=32) had a high level of self-efficacy and 26% (n=13) had a high level of life satisfaction.

**Conclusions:**

This study indicated that young patients transitioning to adult outpatient clinics have good psychological resources and coping mechanisms, which might result in adequate adaptation and adult life satisfaction including future metabolic control. These result also disprove the stereotypes that young people with chronic disease have worse life perspectives when entering adulthood.

## Introduction

In the health care system in Poland, the treatment of patients aged <18 years is provided by pediatric clinics; this also applies to pediatric diabetes care. After becoming 18 years old, a young person with type 1 diabetes mellitus (T1DM) is redirected to adult diabetic care. From the psychological point of view, the moment of transition from adolescence into adulthood is associated with many emotional and social challenges—young people are still in the process of developing their identity and personality and many important decisions concerning their future will need to be made [1]. These changes also concern diabetes; patients have to confront with the need to become responsible for various decisions and duties that so far were more caregiver based. It is necessary to undertake the responsibility for everyday management of diabetes—both emotional and clinical, which may be overwhelming, especially for young people whose parents were either overprotective or not engaged, which is a common issue in families with a child with chronic disease [2-4]. The results of the SEARCH for Diabetes in Youth Study [5] indicated a 2.5-fold increase in the risk of deterioration of metabolic control in a young adult cohort with T1DM leaving pediatric care compared to those under the care of pediatric clinics. A satisfactory transition process is defined based on the achievement of target glycated hemoglobin (HbA_1c_) values, the presence or absence of acute and chronic diabetic complications, and the quality-of-life assessment of young people with T1DM [6,7].

In 2013, the Polish Diabetes Association clinical recommendations for the management of patients with diabetes presented guidelines for transferring patients with type 1 diabetes from pediatric care to adult care [8]. According to the recommendations, every patient transferred to adult medical care should receive a Pediatric Care Information Card containing all relevant information regarding the course of diabetes in a pediatric facility [8]. The existing routines for transfer between pediatric and adult care are not optimal, and structured transition programs may be effective in decreasing the adverse outcomes of that process [9,10]*.* A recent review of transition practices is presented in recommendations by Modrzyńska and Szadkowska [11].

Modifiable factors that were shown to be associated with glycemic control and may play a special role in the transition period are diabetes distress, self-efficacy, and transition readiness [12].

Many of the international publications focus on the difficulties and risks connected with the transition process. The aim of our study was to evaluate the psychological parameters, strengths, and resources of youth with type 1 diabetes during the first visit at an adult outpatient clinic during the transition process.

## Methods

### Psychological Questionnaires

This was a cross-sectional study at 3 diabetes centers. The diabetes centers were tertiary referral centers, which are the biggest in each region, with over 500 patients each from 3 regions in southern Poland.

In all, 50 consecutive young adult patients (center A, n=16; center B, n=21; and center C, n=13) were enrolled during the transition from pediatric care to an adult outpatient clinic (up to 5 young adult patients in each center refused to take part in the study). During the first visit at an adult clinic, after obtaining written consent, a set of psychological questionnaires were given:

1. Personal questionnaire: information about age, body mass, weight, the place of living, the duration of diabetes, the model of treatment, and the type of insulin used.
2. Perceived Stress Scale (PSS-10): a 10-item questionnaire originally developed by Cohen et al [13] that is widely used to assess stress levels in young people and adults aged 12 years and older; it evaluates the degree to which an individual has perceived life as unpredictable, uncontrollable, and overloading over the previous month.
3. Satisfaction with Life Scale (SWLS) [14]: a measure of global life satisfaction; scores on the SWLS correlate moderately to highly with other measures of subjective well-being and correlate predictably with specific personality characteristics.
4. Acceptance of Illness Scale (AIS) [15]: a measure of illness acceptance in any condition; the scale consists of 8 statements describing negative consequences of poor health and limitations imposed.
5. Generalized Self-Efficacy Scale (GSES) [16]: a self-report measure of self-efficacy, which is correlated to emotion, optimism, and work satisfaction; negative correlations were found for depression, stress, health complaints, burnout, and anxiety.
6. Multidimensional Health Locus of Control (MHLC) Scale Form C [17]: based on earlier work with a general Health Locus of Control scale, this is an 18-item scale evaluating health locus of control in 4 dimensions: internal, chance, physicians, and others.
7. Courtauld Emotional Control Scale (CECS) [18]: a commonly used self-report tool for assessing emotional suppression in both clinical and general groups.
8. State-Trait Anxiety Inventory (STAI) [19]: a tool that measures anxiety as a transient and situationally determined state of the individual and a relatively stable personality trait; the STAI consists of 2 subscales, one (X1) measuring state anxiety and the other (X2) measuring trait anxiety; each subscale consists of 20 items that the respondent answers by selecting 1 of 4 precategorized answers.
9. Quality of Life Questionnaire (QoL-Q) Diabetes: a measure of the quality of life for adults with type I diabetes by Speight J et al [20]; the questionnaire is a self-assessment scale composed of 2 parts; the first part measures the quality of life with diabetes in a given (1 of 23) life areas, and second part assesses the importance of each of the 23 aspects of life on a 3-dimension scale.

To compare the results with general healthy population, we used data obtained in validations studies performed on the general Polish population and the general Polish population of patients with diabetes from the Polish Test Laboratory [16-21]. For each of the comparisons, we had specific data: the number of examined participants, mean, SD, age, and sex. A more detailed description of the reference population is presented in the above manuals, separately for each test [16-21].

### Statistical Analysis

We compared the data obtained in the study with the data available from Polish Test Laboratory validation studies for the general healthy population and the general population of persons with diabetes. To compare 2 independent variables, the Student or Welch 2-tailed *t* test for normally distributed (Shapiro-Wilk test) continuous variables was used; otherwise, the Mann-Whitney *U* test was used. To compare 2 dependent groups, the paired 2-tailed *t* test or Wilcoxon signed-rank test, when appropriate, was used. To compare 3 groups, ANOVA or the Kruskal-Wallis test, when appropriate, was used. Correlation between 2 qualitative variables were assessed using Pearson or Spearman correlation, when appropriate. Chi-square test was used to test for associations between categorical variables at 5% significance level. Analyses were performed with R (version 4.2.1; R Foundation for Statistical Computing) and RStudio (version 2022.07.2 Build 576; Posit, PBC).

### Ethics Approval

The study followed the principles of the Declaration of Helsinki and was approved by the Jagiellonian University Bioethics Committee (approval 1072.6120.12.2021 of February 17, 2021). All patients signed informed consent to participate in this study. The study data are anonymously stored (name and surname as initials). There was no additional compensation for the patients.

## Results

From March 2, 2021, to November 21, 2022, during the first visit at an adult care clinic, 50 consecutive young adults (n=28, 56% female) were recruited to complete a set of psychological questionnaires. The mean transition time was 5.6 (range 2-18) months. The mean patients age was 19.2 (SD 1.4) years, with a mean diabetes duration of 9.8 (SD 4.3) years, a mean BMI of 23.5 (SD 3.1) kg/m^2^, and a mean HbA_1c_ level of 7.5% (SD 1.2%). In all, 67% (n=33) of the young adults were not optimally treated (HbA_1c_ >7.0%; Table 1).

Patients came from diverse backgrounds: 36% (n=18) live in villages, 26% (n=13) live in towns with ≤100,000 inhabitants, and 38% (n=19) live in bigger towns. There was no difference between the 3 medical centers and socioeconomic backgrounds regarding the level of all analyzed psychological parameters (all *P*>.05). Patients from Silesia were older than patients from the Lesser Poland and Subcarpathia regions (20.7 vs 18.5 vs 18.5 years, respectively; *P*<.001).

In all, 68% (n=34) of patients were treated with personal insulin pump therapy, whereas 32% (n=16) were treated with multiple daily injections (MDIs). There was no difference regarding the mode of treatment for all analyzed psychological traits, as well as for age, BMI, and diabetes duration.

Most patients (n=32, 64%) had a high level of self-efficacy (n=12, 24% average level and n=6, 12% low level) and an average level (n=21, 42%) of life satisfaction (n=16, 32% low level and n=13, 26% high level). Considering the placement of health locus of control, 62% (n=31) of patients had a high level of belief that control over their own health depends on themselves; 52% (n=26) felt that their own health depends mostly on others, especially medical staff; and 54% (n=27) felt that chance or other external factors have an impact on their health. Deeper analysis of the strength of health locus of control showed that most (n=10, 20%) were of the *undifferentiated-strong* type, 8 (16%) were of the *strong-internal* type, and 7 (14%) were the *magnifying the impact of chance* type. The *increasing the influence of others* type was represented by 4 (8%) patients; and the same number of patients (n=4, 8%) were of the *undifferentiated-weak* type (Table 2).

There was no difference regarding the level of life satisfaction, perceived level of stress, and the levels of both state and trait anxiety between the patients and the general population and the general population of patients with diabetes, apart from a difference between the patients and general population in trait anxiety in the subgroup of male patients older than 18 years. The patients had similar health locus of control and negative emotions control to the general population of patients with diabetes.

The patients had higher levels of suppression of negative emotions—anger, depression, and anxiety—than the age-matched general population. In addition, our population was characterized by a higher acceptance of illness (*P*<.001) and a higher level of self-efficacy (*P*<.052, but this was not statistically significant) compared to the general population and the general population of patients with diabetes (Table 3). A higher HbA_1c_ level correlated with a lower level of self-efficacy (*r*=–0.28; *P*=.051, but this was not statistically significant). No other analyzed psychological traits correlated with HbA_1c_ level.

**Table 1:** Baseline characteristics of the participants.

Variable		Value, mean (SD)	Value, median (IQR)	Value, range
HbA_1c_^a^ level (%)		7.53 (1.15)	7.50 (6.90-8.10)	5.40-12.49
BMI (kg/m^2^)		23.5 (3.1)	23.8 (21.3-25.4)	16.7-29.1
Age (years)		19.2 (1.4)	19.0 (18.0-20.0)	21.0-23.0
Diabetes duration (years)		9.8 (4.3)	10.0 (6.3-13.0)	2.0-17.0
**Psychological characteristics**				
	PSS10^b^ score	18.3 (7.3)	19.0 (13.3-21.8)	5-34
	PSS10 sten^c^ score	6.1 (2.1)	6.5 (4.3-7.0)	3-10.0
	GSES^d^ score	30.1 (4.3)	31 (28.0-33.0)	18.0-38.0
	GSES sten score	6.6 (1.7)	7.0 (6.0-8.0)	2.0-10.0
	STAI^e^ X1 score	39.3 (9.8)	38.0 (32.3-44.8)	20-68
	STAI X1 sten score	5.9 (2.2)	6.0 (4.3-7.8)	1-10
	STAI X2 score	43.8 (10.33)	43 (35.8-49.3)	20.0-67.0
	STAI X2 sten score	6.1 (2.3)	6.0 (4.0-8.0)	1-10
	SWLS^f^ score	19.8 (5.9)	21 (15.3-23.8)	6-32
	SWLS sten score	5.4 (2.1)	6.0 (4-6.8)	1-10
	AIS^g^ score	30.7 (7.5)	33.5 (26.3-36.8)	14-40
	CECS^h^ score	56.52 (12.79)	56.5 (47.0-63.8)	31-84
	CECS Anger score	18.1 (5.3)	18.0 (14.3-22.0)	9-28
	CECS Depression score	19.9 (5.1)	20.0 (16.0-24.0)	10-28
	CECS Anxiety score	18.6 (5.2)	19.0 (15.0-22)	7-28
	MHCL^i^ Scale Internality score	26.9 (4.3)	27.0 (23.3-30.0)	16-34
	MHCL Scale Doctors and Other (powerful) score	23.9 (4.5)	25.0 (22.0-26.0)	13-33
	MHCL Scale Chance score	19.3 (4.9)	20.0 (15.5-23.0)	10-28
	QoL-Q score	197.9 (50.1)	201.0 (160.0-229.0)	98-305

^a^HbA_1c_: glycated hemoglobin.

^b^PSS-10: Perceived Stress Scale.

^c^Sten: standard ten (standardized scores from 1-10).

^d^GSES: Generalized Self-Efficacy Scale.

^e^STAI: State-Trait Anxiety Inventory.

^f^SWLS: Satisfaction with Life Scale.

^g^AIS: Acceptance of Illness Scale.

^h^CECS: Courtauld Emotional Control Scale.

^i^MHLC: Multidimensional Health Locus of Control.

**Table 2:** Classification of types of health locus of control.

Type	Patients (N=50), n (%)
Undifferentiated-strong	10 (20)
Strong-internal	8 (16)
Magnifying type impact of chance	7 (14)
Increasing the impact of chance	6 (12)
Magnifying influence of others	6 (12)
Strong-external	5 (10)
Increasing the influence of others	4 (8)
Undifferentiated-weak	4 (8)

**Table 3:** Outcomes of the psychological tests—comparison of the study participants with the general population.

Questionnaire				General healthy population				Patients with T1DM^a^					*P* value
			Value, n		Value, mean (SD)	Value, n			Value, mean (SD)				
**STAI^b^ score**													
	**X1 subscale**												
		Male patients aged 17-18 years	71		37.11 (9.20)	12			38.08 (10.83)		.98		
		Female patients aged 17-18 years	150		36.99 (8.30)	11			36.45 (5.59)		.77		
		Male patients aged 21-40 years^c^	89		37.25 (8.65)	10			40.20 (10.67)		.40		
		Female patients aged 21-40 years^c^	90		36.80 (8.37)	17			36.79 (8.12)		>.99		
	**X2 subscale**												
		Male patients aged 17-18 years	71		39.08 (9.01)	12			43.64 (10.24)		.15		
		Female patients aged 17-18 years	150		41.92 (8.62)	11			41.60 (8.06)		.90		
		Male patients aged 21-40 years^c^	89		39.46 (7.06)	10			46.90 (9.27)		.01		
		Female patients aged 21-40 years^c^	90		43.27 (8.06)	17			41.52 (8.61)		.44		
**GSES^d^ score**													
	Overall			496	27.32 (5.32)		50			30.06 (4.34)		<.001	
	General population of patients with diabetes			70	28.34 (5.35)		50			30.06 (4.34)		.052	
**PSS-10^e^ score**													
	Overall			1830	16.62 (7.50)		50			18.34 (7.30)		.10	
	General population of patients with diabetes			70	17.5 (5.92)		50			18.34 (7.30)		.50	
**SWLS^f^ score**													
	Overall			555	20.37 (5.32)		50			19.78 (5.93)		.50	
	General population of patients with diabetes			70	20.34 (5.79)		50			19.78 (5.93)		.61	
**AIS^g^ score**													
	General population of patients with diabetes			70	24.81 (7.09)		50			30.72 (7.48)		<.001	
**MHLC^h^ Scale Form C score**													
	**Internality subscale**												
		Patients aged 18-25 years	211		28.55 (4.01)	50			26.88 (4.31)		.01		
		General population of patients with diabetes	70		25.77 (6.28)	50			26.88 (4.31)		.25		
	**Doctors and Other (powerful) subscale**												
		Patients aged 18-25 years	211		18.70 (4.62)	50			23.94 (4.46)		<.001		
		General population of patients with diabetes	70		25.59 (6.83)	50			23.94 (4.46)		.11		
	**Chance subscale**												
		Patients aged 18-25 years	211		15.76 (4.82)	50			19.32 (4.89)		<.001		
		General population of patients with diabetes	70		20.14 (6.46)	50			19.32 (4.89)		.43		
**CECS^i^ score**													
	**Total**												
		Patients aged 20-30 years	264		48.58 (10.62)	50			56.52 (12.79)		<.001		
		General population of patients with diabetes	70		55.77 (9.86)	50			56.52 (12.79)		.73		
	**Angel Control subscale**												
		Patients aged 20-30 years	264		15.29 (4.72)	50			18.06 (5.34)		<.001		
		General population of patients with diabetes	70		17.85 (4.27)	50			18.06 (5.34)		.81		
	**Depression Control subscale**												
		Patients aged 20-30 years	264		16.18 (4.22)	50			19.88 (5.05		<.001		
		General population of patients with diabetes	70		19.17 (4.55)	50			19.88 (5.05)		.43		
	**Anxiety Control subscale**												
		Patients aged 20-30 years	264		17.12 (4.54)	50			18.58 (5.23)		.06		
		General population of patients with diabetes	70		18.75 (3.60)	50			18.58 (5.23)		.84		

^a^T1DM: type 1 diabetes mellitus.

^b^STAI: State-Trait Anxiety Inventory.

^c^Diabetes group included patients aged 19-20 years.

^d^GSES: Generalized Self-Efficacy Scale.

^e^PSS-10: Perceived Stress Scale.

^f^SWLS: Satisfaction with Life Scale.

^g^AIS: Acceptance of Illness Scale.

^h^MHLC: Multidimensional Health Locus of Control.

^i^CECS: Courtauld Emotional Control Scale.

## Discussion

In this cross-sectional study, we evaluated the strengths and resources of patients with T1DM during the transition to adult diabetes care; thus, we examined self-efficacy, the acceptance of illness, satisfaction with life, the level of anxiety, stress, health locus of control, and negative emotions control among youth with type 1 diabetes during the first visit at an adult outpatient clinic. The mean time of transition was around the recommended 6 months.

The main findings indicate that the young adults in this study had a high level of resources, allowing them to accept the illness (at a higher level than the general population of patients with diabetes), and had a higher level of self-efficacy compared to general population and the general population of patients with diabetes (but this finding was not statistically significant). Mental health is an important factor of diabetes care; it is in fact a prerequisite for coping effectively with the 24/7 self-care demands of T1DM and, thus, for achieving and maintaining optimal glycemic outcomes that minimize the risk of developing complications. On the other hand, research indicates that dealing with diabetes for many years, especially since early childhood, may result in diabetes distress or diabetes burnout and that psychological support is needed at various stages of development [22,23]. Furthermore, studies show higher prevalence of depression, eating disorders, and anxiety disorders in population of patients with T1DM compared with the general population, and these comorbidities often requiring specialist treatment [22]. Patients with T1DM who experience mental disorders often experience difficulties in diabetes self-management, which is associated with elevated HbA_1c_ levels and a higher complication risk, also during the transition period. It has been indicated that the level of depression negatively correlates with the level of acceptance of the disease and life satisfaction, whereas the level of anxiety correlates only with the level of acceptance of the disease [23]. In patients with type 2 diabetes, the level of disease acceptance is an independent predictor of adherence [24]. Some studies also suggest that there is a difference in accepting therapy between users of continuous subcutaneous insulin infusion and MDIs [25]. Quality of life and illness acceptance were found to be strongly related. Patients with chronic peripheral diabetic neuropathy express lower degrees of acceptance of their illness than patients with diabetes without peripheral diabetic neuropathy, but in our population, we did not assess the presence of late diabetes complications [26]. The knowledge of this correlation between mental health and diabetes control is important to providing the proper care and support for young people with diabetes. However, these results do not mean that every young patient with diabetes experiences mental health difficulties and that, if those difficulties occur, they must be a permanent obstacle in obtaining life goals by the young person.

For many years, there has been a tendency to create stereotypes concerning patients with chronic illnesses, which assume that because of their medical condition they are less ready to undertake various social and family roles. There was a conviction, often communicated to the young patients with T1DM, that they should not set too ambitious goals in their lives and that they should focus mostly on diet and glycemic control instead of planning their future in terms of their dreams and goals. Many of these stereotypes were also present in society, and as a consequence, some young people with T1DM were rejected from particular work positions; although there were no clinical indications for such limitation, many were also afraid to reveal their diabetes openly for fear that they could be labelled, rejected, and stigmatized [27-29]. This attitude was often harmful for patients with T1DM who in fact coped well with their diabetes, as well as to those who experienced some difficulties and as a result, instead of asking for professional help and support, preferred to hide their problems and to withdraw from life challenges [30].

Our study indicated that most of the young patients with T1DM have a high level of self-efficacy, are able to deal with everyday stressors, do not experience elevated level of state anxiety, and are ready to undertake the challenges connected with everyday diabetes control on their own. To some extent, this may cause them to be ineligible for psychological treatment or unwilling to seek professional support, because they wish to self-manage and solve their problems on their own or have a fear of being stigmatized; however, in general, it shows that their level of psychological resources is not lower than those of the general population. Even patients who have already a long history of diabetes and experienced various situations with the treatment have resources that make them ready to start their adult life with every chance to succeed in their life goals. The only exception from this finding was the level of trait anxiety, which was elevated in the subgroup of male patients older than 18 years. This shows that throughout the many years of examining the development of this population of patients, although we observed that they may have tendency to suppress their negative emotions too much, they have experienced stressful situations that they deal with on a regular basis and learned to cope effectively. We also indicate that the patients are focused on their diabetes more than patients with diabetes from the general population, placing the responsibility for their health in their own coping strategies as well as in medical staff. Although all the young people are, in general, in challenging life situations that are connected with many changes, including the change of the diabetologist and treatment team, they seem to be well prepared and ready to pick up their new tasks. The fact that the groups from 3 centers presented similar results allow us to speculate that this observation could be generalized to the population of present young people with T1DM in Poland, although the examined sample was not very large.

However, it does not mean that these patients do not experience any difficulties. As indicated above [22,23,31] and also in our own previous studies [32], many patients with T1DM experience symptoms of emotional difficulties and mental health problems. This should never be neglected and must be strictly monitored while offering proper help when needed. However, in this study, we did not want to focus on psychopathology, which is described in many publications [33-39]. Our goal was to challenge the negative stereotypes and stigma concerning the life perspectives of young people with T1DM and to examine their coping possibilities during the transition from adolescence into adulthood in terms of both the possible cooperation with the new medical team and, more broadly, their psychological readiness to start a full and fruitful life.

The significance of quality of life is often stressed in guidelines concerning treatment for T1DM; however, there is still a need to prepare more precise indications for family members of children with type 1 diabetes about adequate coping strategies. It is now widely recognized that a patient with good control of their diabetes is a person who not only has proper metabolic control but also has good psychological well-being and is able to have a satisfactory life in many of its aspects [40,41]. The mean quality-of-life score obtained in this study was similar to our previous publications for older patients [42].

It has been shown that insulin pump treatment may be associated with behavior change and less hypoglycemia in comparison to MDIs [43,44]. One may wonder if this mode of treatment also influences the psychological resources. In our study, we did not find any difference regarding the mode of treatment for all analyzed psychological traits. Additionally, continuous glucose monitoring system use may change patients’ hypoglycemia avoidance behaviors and, when used in combination with an insulin pump, may improve treatment satisfaction, but we did not assess this effect in this study [45-48].

In general, the transition process took around 6 months, which is in line with the current guidelines [6,8]. The mean age of the transition in our study was 19.2 (SD 1.4) years. One center has older patients than the other 2. It may be advisable to prolong the transition visit from the age of 18 to 19 years and take this additional year to prepare for this process [8].

This study has limitations that should be acknowledged. The number of patients with T1DM examined per center in our study was relatively low. After more than 1.5 year of recruitment, we decided to cease the continuation of the study as few young adults were in the transition phase. Additionally, we did not assess the presence of patients’ late diabetes complication; however, this group was characterized by relatively short diabetes duration (mean 9.8, SD 4.3 years). Part of the study was carried out during the COVID-19 pandemic period, and it was impossible to assess the probable impact of the factors connected with the pandemic (lockdown, the change of daily routines, limited access to face-to-face consultations, stress connected with the possibility of being infected, coronavirus infection, etc) on the evaluated psychological parameters. We also did not assess continuous glucose monitoring systems use, which may have had an impact on higher psychological resources in our patients. Finally, our study was performed only once at the first admittance visit, and we did not collect data during follow-up visits.

In conclusions, this study indicated that young patients transitioning to adult outpatient clinics have good psychological resources and coping mechanisms, which might result in adequate adaptation and adult life satisfaction including future metabolic control. The results also contradict the stereotypes that young people with chronic disease have worse life perspectives when entering adulthood. Nevertheless, monitoring the mental health of patients with T1DM should be an ongoing part of diabetes care, even in patients who currently have good psychological resources.

## Abbreviations

AIS: Acceptance of Illness Scale
CECS: Courtauld Emotional Control Scale
GSES: Generalized Self-Efficacy Scale
HbA_1c_: glycated hemoglobin
MDI: multiple daily injection
MHLC: Multidimensional Health Locus of Control
PSS-10: Perceived Stress Scale
STAI: State-Trait Anxiety Inventory
SWLS: Satisfaction with Life Scale
T1DM: type 1 diabetes mellitus
QoL-Q: Quality of Life Questionnaire

## Data Availability

The data sets generated during and analyzed during the current study are available from the corresponding author on reasonable request.
